# Comparative efficacy on dogs of a single topical treatment with the pioneer fipronil/(S)-methoprene and an oral treatment with spinosad against *Ctenocephalides felis*

**DOI:** 10.1051/parasite/2011184325

**Published:** 2011-11-15

**Authors:** F. Beugnet, V. Doyle, M. Murray, K. Chalvet-Monfray

**Affiliations:** 1 Merial S.A.S. 29, avenue Tony Garnier 69007 Lyon France; 2 Charles River Laboratories Preclinical Services Ireland Ltd, Carrentrila, Ballina Co. Mayo Ireland; 3 Merial Limited 3239 Satellite Bvd Duluth GA30096 USA; 4 Biomathematics Unit, Veterinary Faculty 1, avenue Bourgelat 69280 Marcy L’Étoile France

**Keywords:** fipronil/(S)-methoprene, spinosad, *Ctenocephalides felis felis*, duration of efficacy, flea egg, FRONTLINE^®^ PLUS, COMFORTIS^®^, fipronil/(S)-méthoprène, spinosad, *Ctenocephalides felis felis*, rémanence, oeuf de puce, FRONTLINE^®^ PLUS, COMFORTIS^®^

## Abstract

In the study reported here, the pioneer fipronil/(S)-methoprene topical product (FRONTLINE^®^ PLUS, Merial Limited, Duluth, GA) was compared to the oral spinosad product (COMFORTIS^®^ Elanco, Greenfield, IN) for efficacy against adult fleas and preventing egg production. The product presentations, doses and labelling were the one applicable in the USA. Using a standard protocol, 200 cat fleas of mixed sex were applied to dogs on Days 1, 7, 14, 21, 28, 35, and 42. Dogs were combed to remove fleas 24 hours post-infestation, the fleas were counted, collected, and then reapplied to each dog following completion of their respective count. At 48 hours post-infestation, comb counts were performed and fleas were removed. No fleas were collected from any dog in the fipronil/(S)-methoprene group at any 24 or 48 hours post-infestation assessment throughout the six weeks study, yielding a preventive efficacy of 100%. For the spinosad treatment, efficacy was 100% at 24 hours and 48 hours through Day 16, and thereafter declined. The results observed in the spinosad-treated dogs were highly variable between animals. At the 24 and 48 hours counts following the Day 21 infestation, only five of eight spinosad-treated dogs (62.5%) were flea-free. Following the Day 28 infestation, spinosad efficacy fell to 85% and 89%, for the 24 hours and 48 hours counts, and only two dogs (25%) were flea free, compared to 100% flea-free dogs in the fipronil/(S)-methoprene group. No fleas were collected from the fipronil/(S)- methoprene treated dogs throughout the entire study, therefore, no eggs were collected at any time from any dog in the group. However, in the spinosad group adult fleas were found on dogs starting on Day 21 and by Day 30, 42 eggs were collected from one dog that had 107 adult fleas counted at 48 hours. At Day 37 and Day 49, more than 100 eggs were collected from each dog in the spinosad-treated and control groups.

## Abstract (fr)

Dans l’étude ici présentée, le produit topique princeps à base de fipronil/(S)méthoprène (FRONTLINE^®^ PLUS, Merial Limited, Duluth, GA) a été comparé au produit oral à base de spinosad (COMFORTIS^®^ Elanco, Greenfield, IN) en ce qui concerne l’efficacité anti-puces et la prévention de la production d’oeufs de puces. Les présentations, dosages et notices d’utilisation qui ont été suivies sont celles de ces produits commercialisés aux États-Unis. Utilisant un protocole classique, 200 puces adultes mâles et femelles ont été déposées sur des chiens aux jours 0, 7, 14, 21, 28, 35 et 42. Les chiens ont été peignés pour collecter les puces 24 heures après chaque infestation. Ces dernières ont été comptées puis ont été redéposées sur les chiens. 48 heures après chaque infestation, les chiens ont été de nouveau peignés et les puces collectées définitivement. Aucune puce n’a été récupérée sur aucun des chiens traité par le fipronil/(S)-méthoprène à 24 heures ou 48 heures après infestation pendant les six semaines de l’étude, d’où une efficacité préventive de 100 %. En ce qui concerne le groupe traité par le spinosad, l’efficacité préventive a été de 100 % à 24 et 48 heures jusqu’au jour 16, et a ensuite diminué. Les résultats ont été très variables selon les chiens dans ce groupe. À 24 et 48 heures après l’infestation du jour 21, seulement cinq des huit chiens (62,5 %) n’avaient pas de puces. Après l’infestation du jour 28, l’efficacité moyenne tombait à 85 % à 24 heures et 89 % à 48 heures, et seulement deux chiens sur huit (25 %) n’avaient pas de puces, contre 100 % dans le groupe traité fipronil/(S)-méthoprène. Aucun oeuf de puces n’a été collecté durant les six semaines de l’étude sur les chiens du groupe traité par le fipronil/(S)-méthoprène. Dans le groupe traité par le spinosad, des puces adultes sont observées sur les chiens après l’infestation du jour 21, et au jour 30, 42 oeufs ont été récupérés sur un chien qui avait 107 puces comptées à 48 heures. Aux jours 37 et 49, plus de 100 oeufs de puces ont été collectés sur une période de 12 heures sur chaque chien du groupe spinosad, ainsi que sur tous les chiens du groupe contrôle.

Dogs are infested by two flea species: the cat flea, *Ctenocephalides felis felis*, which is considered to be the predominant flea species found on dogs and cats worldwide (Durden *et al*., 2005; Farkas *et al*., 2009; Gracia *et al*., 2007), and the dog flea, *Ctenocephalides canis*, generally regarded as less important (Beck *et al*., 2006; Dryden & Rust, 1994; Franc *et al*., 1998). Fleas are responsible for various clinical signs, including pruritus, alopecia, anaemia, seborrhoea, and development of an allergies (Flea Allergy Dermatitis). Fleas are also the carrier of pathogens, including *Rickettsia felis*, *Bartonella henselae*, and *Mycoplasma-Haemoplasma* and the parasite, *Dipylidium caninum*. Due to their low host-specificity, fleas easily infest numerous species of wild animals, feral dogs and cats, pets and will feed on pet owners. The goals of effective flea control are to provide relief to the pet and eliminate infestation of the premises (Dryden, 2009). Controlling flea infestations is mainly based on the regular administration of flea adulticides on pets, and the use of insect growth regulators (IGR) (Franc & Cadiergues, 1995; Jacobs *et al*., 2001). Available insecticides are represented by many classes with various modes of action (IRAC, 2010). The majority of available treatments for dogs and/or cats are topical spot-on applications (Barnett *et al*., 2008; Cadiergues *et al*., 2001; Rust *et al*., 2007; Young *et al*., 2004). The active molecules of these topical preparations are absorbed and act systemically, or spread on the skin and act by direct contact with the arthropods (Cochet *et al*., 1997). A few products are given orally like lufenuron, nitenpyram, and more recently spinosad (Dobson *et al*., 2000; Kirst, 2010; Robertson-Plouch *et al*., 2008).

The most effective animal-based flea control strategies need to provide: 1 – a sustained activity and speed of kill on adult fleas during a known period (at least a month); and 2 – the control of immature stages by inhibiting the production of eggs and/or the development of flea eggs into larvae, pupae and newly emerged fleas (Dryden, 2009; Dryden *et al*., 2000; Beugnet & Franc, 2010). Failure to kill all fleas before they can reproduce in the absence of an insect growth regulator can permit the persistence of a premise flea infestation.

The aim of this trial was to study and compare the level and duration of efficacy of the topical formulation of fipronil/(S)-methoprene (FRONTLINE^®^[Fn FN2] PLUS, Merial Limited, Duluth, GA) and the oral formulation of spinosad (COMFORTIS^®^ Elanco, Greenfield, IN) against adult fleas on dogs, and to assess their respective efficacy for controlling flea egg production. Fipronil is a phenylpyrazole acting by contact on arthropods, binding and blocking GABA and glutamategated chloride channels on the arthropod nerve cells and inducing the parasite death. Spinosad is a mixture of spinosyns A and D, belonging to the spinosyn macrocyclic lactone group, and acting on insects through ingestion by stimulating the nicotinic acetylcholine receptors of insect nerve cells.

## Materials and Methods

### Study Design

This study was a negative controlled efficacy study using a randomized block design where blocks were based on pre-treatment flea counts. Three groups of eight dogs were formed: untreated control, fipronil/(S)-methoprene treated group and spinosad treated group. 30 healthy Beagle dogs were used, 15 males and 15 females, between eight to 12 months old. To qualify for inclusion, dogs could not have been treated with an ectoparasiticide within three months prior to the start of the study. Animals were managed similarly and with due regard for their well-being. Animals were handled in compliance with the relevant Institutional Animal Care and Use Committee approvals and any other, *e.g.* national or local, applicable regulations and requirements.

All dogs were shampooed, according to the Schedule of Operations, with a non-insecticidal shampoo for approximately five minutes each prior to or on Day -20. Dogs were pre-infested with 200 (± 5) adult unfed *C. felis* once, on Day -16, for selection purposes. Three dogs of each sex with lowest flea counts were removed. Dogs were treated on Day 0, and post treatment, infestations were performed on Days 1, 7, 14, 21, 28, 35, and 42 using 200 (± 5) adult unfed *C. felis*, and counts were performed at 24 and 48 hours post-infestation.

Adult unfed *C. felis* belonging to the CRL strain (Charles River Laboratories, Ireland) were used for infestations. These fleas are not known to be tolerant to any ectoparasiticide. The study was conducted such that all personnel involved in parasite infestation counting and evaluation of results were blinded to the treatment.

### Treatment Administration (Table I)

Dogs were weighed between Day -3 and Day -1 for dosage determinations. When the weights were not a whole kg, they were rounded up to the next whole kg.

**Table I.** Dosage of dogs in the treated groups.

	Bodyweight range – kg (lbs)	Pipette size	Pipette volumes – mL	Tablet size – mg
Frontline^®^ Plus treated dogs	Up to 10 (22 lbs)	S	0.67	
	10.1–20 (23–44 lbs)	M	1.34	

Comfortis^®^ treated dogs	9.1–18.1 (20.1-40 lbs)			560

**Table II.** Number of fleas counted at 24 hours and 48 hours post-infestation, from Day 21 infestation to Day 42 infestation. Calculation of the % of flea free dogs and comparison between the two treated groups.

Groups	Dog tag number	Day 22**	Day 23	Day 29	Day 30	Day 36	Day 37	Day 43	Day 44
Untreated	58091	133	124	149	127	128	135	137	126
	27351	125	103	71	87	119	126	147	155
	40061	135	138	118	124	155	159	121	141
	43861	156	164	132	114	107	141	150	132
	22774	137	121	133	116	118	106	138	133
	39545	117	111	104	106	111	123	152	143
	94289	161	123	132	130	142	128	153	147
	28632	133	154	102	102	141	132	136	144

Frontline^®^ Plus	38603	0	0	0	0	0	0	0	0
	38793	0	0	0	0	0	0	0	0
	35092	0	0	0	0	0	0	0	0
	67804	0	0	0	0	0	0	0	0
	13789	0	0	0	0	0	0	0	0
	42356	0	0	0	0	0	0	0	0
	38257	0	0	0	0	0	0	0	0
	37299	0	0	0	0	0	0	0	0
	
	% Flea free dogs	100 %*	100 %*	100 %*	100 %*	100 %*	100 %*	100 %*	100 %*

Comfortis^®^	89565	0	0	0	0	59	48	118	112
	41363	13	11	87	79	75	89	180	179
	33824	0	0	0	0	74	67	94	77
	02777	0	0	32	22	90	73	127	125
	44355	17	6	57	56	116	113	105	107
	44259	0	0	19	3	0	0	122	112
	42352	0	0	31	22	23	42	134	141
	27347	48	15	115	107	106	104	125	168
	
	% Flea free dogs	62.5 %*	62.5 %*	25 %*	25 %*	12.5 %*	12.5 %*	0 %*	0 %*

* Significant difference between the two treatment groups at all time points after Day 21 infestation at p < 0.05.

** Table starting at Day 22 because no fleas were counted in any of the treated group the weeks before.

Dogs in Treatment Group 1, negative control, were untreated.

Dogs in Treatment Group 2 were treated with the appropriate pipette size of FRONTLINE^®^ PLUS spoton. For treatment administration, the total volume was applied on one spot placed on the midline of the neck. The hair was parted at the base of the neck, between the shoulder blades, until the skin was visible. The tip of the pipette was placed on the skin and squeezed to empty its contents directly onto the skin. The dogs treated with FRONTLINE^®^ PLUS weighed from 10 to 13.3 kg. They were all treated with the 1.34 mL pipette except the 10 kg dog with the 0.67 mL pipette.

Dogs in Treatment Group 3 were treated with the appropriate chewable tablet of COMFORTIS^®^, using the US presentations and labeling. The tablet was administered with food as recommended by the manufacturer. Following treatment on Day 0, all dogs were observed hourly (± 30 minutes) for four hours after the last animal was treated. The dogs treated with COMFORTIS^®^ weighed from 10.4 to 15.8 kg. They were all treated with the 560 mg tablet. The minimal treatment dose is 30 mg/kg in the USA and 45 mg/kg in Europe. Except two dogs treated at 35.44 mg/kg and 38.55 mg/kg, all the others received a dose over the minimum European recommended dose of 45 mg/kg.

### Specification of Study Variables

#### • Adult flea count

Fleas were comb-counted initially at 24 hours postinfestation, then replaced on the dog, and then counted and removed at 48 hours post-infestation on all dogs.

#### • Egg count

Flea eggs were collected overnight for a 12 hours period from 36 to 48 hours post infestation. The dogs were place in a cage with a grille at the bottom and a dark plate below the grille. The flea eggs are small, oval and white, measuring 0.4 × 0.6 mm. When the 24 hours adult flea counts were found to be positive, those dogs were subsequently placed in cages for flea egg collection. It was predetermined that egg counts at or higher than 100 eggs per dog within a 12 hours period would be sufficient enough to ensure the animal’s environment would be continuously contaminated. Egg totals less than 100 were recorded, but when the egg counts reached or exceeded the threshold of 100 flea eggs within a 12 hours period, the count was entered as a maximum of 100 eggs.

### Data Analysis

#### • Adulticidal efficacy

Counts of live adult fleas were transformed to the natural logarithm of (count + 1) for calculation of geometric means by treatment group at each time point. Percent reduction from the negative control mean was calculated using the formula [(C - T) / C] × 100, where C = geometric mean for the control group and T = geometric mean for the treated group. Arithmetic means were also calculated.

#### • Statistical analysis

The three treatment groups were initially tested using the Kruskal-Wallis rank sum test. Because all Kruskal- Wallis rank tests were significant, a non parametric multiple comparison test (Pairwise Wilcoxon Rank Sum Test) was done at each date. Thus for each date, the test compared Control *vs* FRONTLINE^®^ PLUS, Control *vs* COMFORTIS^®^, and COMFORTIS^®^ *vs* FRONTLINE^®^ PLUS.

## Results

### Adulticidal Efficacy (Tables II and III)

No fleas were collected in the fipronil/(S)- methoprene treated group on any dog at any time during the six weeks of the trial. The preventive efficacy was 100 %, and all dogs remained free of fleas (Tables II and III).

**Fig 1.**
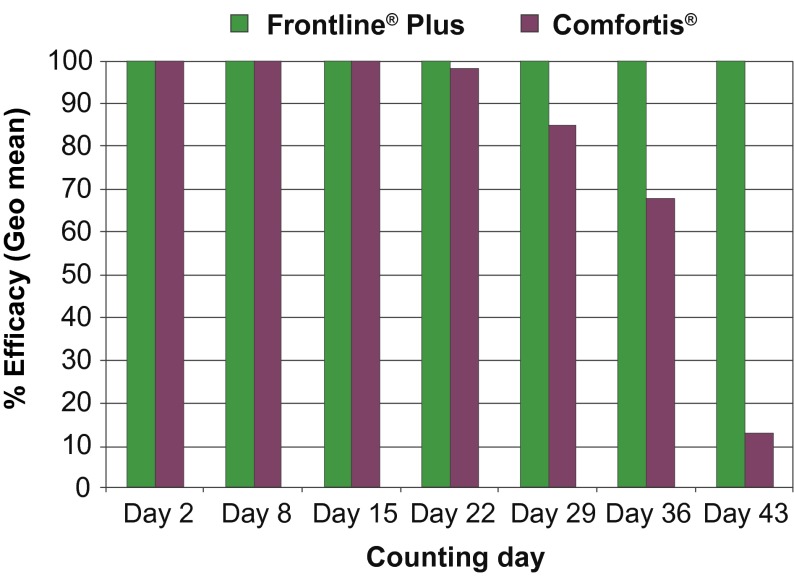
Comparative % of adult flea efficacy in the two treated groups at 24 hours counts.

In the group treated with spinosad, efficacy was 100 % during the first two weeks (from Day 1 to Day 14 challenge), but for the Day 28 infestation efficacy fell to 85 % at the 24 hours count and to 89 % at the 48 hours count (Table III, Fig. 1). The geometric and arithmetic means were significantly different between the spinosad and the fipronil/(S)-methoprene groups following the weekly infestations on Day 28, Day 35 and Day 42 at both 24 hours and 48 hours (p < 0.01 at each time counts). There was no significant difference between the spinosad group and the untreated control group at the 48 hours count following the Day 42 infestation.

**Table III.** Compared killing efficacies of the two treatments on adult fleas at 24 and 48 hours counts.

		Day 2	Day 8	Day 15	Day 22	Day 29	Day 36	Day 43
% Efficacy at 24 hours counts	Frontline^®^ Plus	100	100	100	100	100	100	100,0
	Comfortis^®^	100	100	100	98.4	85.0*	67.8*	12.6*

% Efficacy at 48 hours counts	Frontline^®^ Plus	100	100	100	100	100	100	100,0
	Comfortis^®^	100	100	100	98.9	89.0*	67.9*	11.5*

* Significant difference between the two treatment groups at Day 29, Day 36 and Day 43 at p < 0.01.

**Fig 2.**
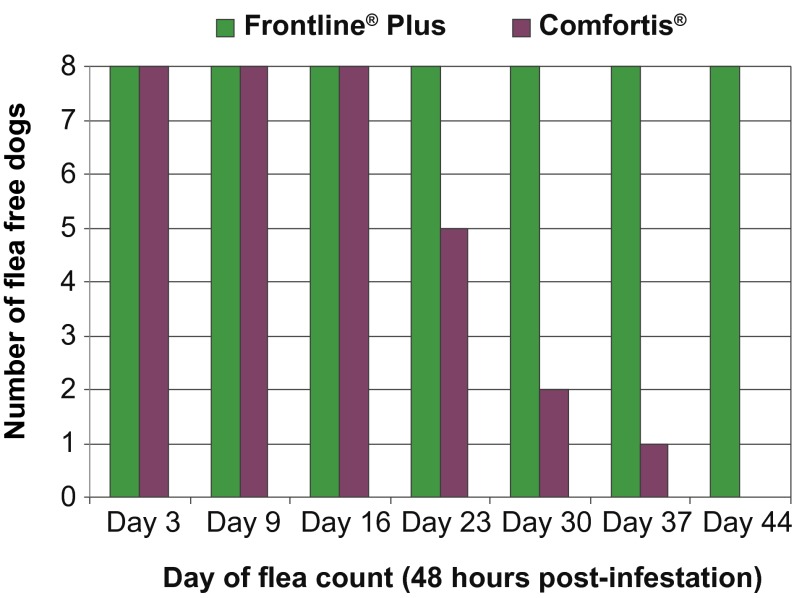
Comparative number of flea free dogs in the two treated groups at 48 hours counts.

**Fig 3.**
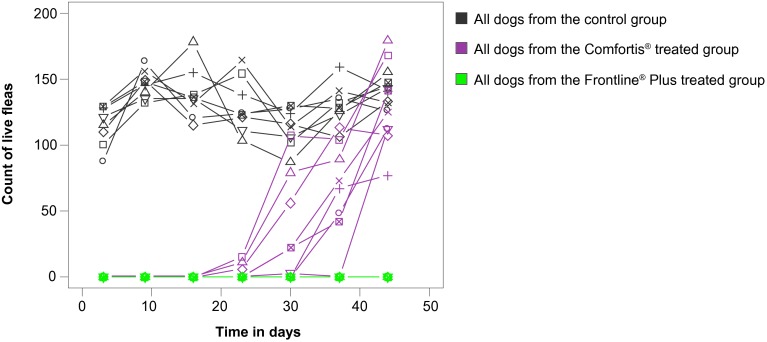
– Distribution of the number of live fleas per each dog at each counting point. One line represents the data observed in the same dog.

Moreover, the results in the spinosad treated group were highly variable between dogs (Table II; Figs 2 and 3). Following the Day 21 infestation, only 5/8 (62.5 %) dogs remained free of fleas at 24 and 48 hours in the spinosad group, and only 2/8 (25 %) of them were still free of fleas following the Day 28 infestation.

### Egg Collections (Table IV)

No eggs were collected from the fipronil/(S)-methoprene group at any time on any dog. A single application of the fipronil/(S)-methoprene topical controlled all flea stages during the entire six weeks. For the infested dogs that produced flea eggs, eggs were randomly collected until a target number of 100 per each dog. In the control group, 100 eggs were collected from each dog at every collect time.

**Table IV.** Number of egg collections overnight per each dog from Day 9 to Day 44.

		Number of eggs collected overnight with a threshold at 100 eggs/dog					
Groups	Dog tag number	Day 9	Day 16	Day 23	Day 30	Day 37	Day 44
Untreated	58091	100	100	100	100	100	100
	27351	100	100	100	100	100	100
	40061	100	100	100	100	100	100
	43861	100	100	100	100	100	100
	22774	100	100	100	100	100	100
	39545	100	100	100	100	100	100
	94289	100	100	100	100	100	100
	28632	100	100	100	100	100	100

Frontline^®^ Plus	38603	0	0	0	0	0	0
	38793	0	0	0	0	0	0
	35092	0	0	0	0	0	0
	67804	0	0	0	0	0	0
	13789	0	0	0	0	0	0
	42356	0	0	0	0	0	0
	38257	0	0	0	0	0	0
	37299	0	0	0	0	0	0

Comfortis^®^	89565	0	0	0	0	100	100
	41363	0	0	0	0	100	100
	33824	0	0	0	0	100	100
	02777	0	0	0	0	100	100
	43355	0	0	0	0	100	100
	44259	0	0	0	0	80	100
	42352	0	0	0	0	100	100
	27347	0	0	0	42	100	100

In the spinosad group, 42 eggs were collected from one dog following the Day 28 infestation. That same dog had 107 adult fleas counted at 48 hours. Following the Day 35 and Day 42 infestations, the maximum threshold of 100 eggs collected was exceeded on all dogs in the spinosad treated group.

## Discussion

This well controlled study confirmed both the high efficacy and the long duration of efficacy of the pioneer fipronil/(S)-methoprene (FRONTLINE^®^ COMBO/FRONTLINE^®^ PLUS, Merial Limited, Duluth, GA) topical formulation on dogs with at least six weeks of 100 % efficacy. Similar results were previously obtained in other trials on both dogs and cats (Cadiergues *et al*., 2001; Franc *et al*., 2007; Schenker *et al*., 2003; Young *et al*., 2004). The results of this study and those of previous studies also demonstrate the consistency of control provided by this formulation from one dog to the next. Due to the high level of efficacy and speed of kill of the fipronil, no eggs were collected on the dogs at any time of the study. It was previously shown that if any newly acquired fleas survive long enough to produce eggs, the eggs laid would be sterilized by (S)-methoprene (Young *et al*., 2004).

In this study, the treatment with spinosad provided complete flea control for only 16 days after treatment. After the D21 infestation, only five of the eight (62.5 %) spinosad-treated dogs were flea-free at both the 24 hours and 48 hours counts, and after the Day 28 infestation only two of the eight dogs (25 %) remained flea-free at both the 24 hours and 48 hours counts.

Every effort was made in the study design to ensure consistency of the animals; dogs were selected from the same breed, same age and similar weight ranges and were maintained under essentially identical management conditions for assessments, feeding, watering and handling. The consistency of the flea burdens observed on the control dogs attests to both the consistency of animals and management practices.

The variable and declining flea-killing efficacy of orally administered spinosad were observed in other post-approval laboratory studies. In one report (Blagburn *et al*., 2010), efficacy studies using the oral spinosad formulation were conducted at research laboratories in the United States and in Ireland, with each site using its own flea strain. Purpose-bred Beagle dogs that were 14 to 16 weeks old were used. Data combined from the three study sites revealed that 48 hours pulicidal efficacies for oral spinosad were 100 % through Day 9, thereafter declining to 85 % (Day 16), 63 % (Day 23), and 2 % (Day 30). There were no statistical differences (p > 0.05) in the number of live fleas recovered from the dogs treated with oral spinosad compared to untreated control dogs on study Days 22 and 29 for two study sites and on Day 29 for the third site.

In a study conducted at another research laboratory, purpose-bred mongrel dogs that were eight to 11 months old were used, and they were treated with oral spinosad on Day 0 according to label directions (Dryden *et al*., 2010). 100 cat fleas of the KS1 flea strain were applied to each dog on Days -2, 7, 14, 21, and 28 after treatment, and the fleas were removed with a flea comb six and 24 hours postinfestation. Oral spinosad provided at least 93.9 % efficacy at six hours post-infestation through Day 14, dropping to 54.9 % efficacy on Day 21 and 35 % on Day 28. Efficacy on Day 29 (24 hours post-infestation) was 22 %, and the geometric mean flea counts on treated dogs were not statistically different from untreated controls.

The unpredictably variable efficacy provided by oral spinosad after 16 days post-treatment may be due to different absorption, metabolism and elimination kinetics between dogs. Orally administered spinosad acts by the systemic route after absorption from the gastrointestinal tract (Kirst, 2010). Data from the Comfortis Food and Drug Agency (FDA) Freedom of Information (FOI) summary showed a variable rate of elimination of spinosyns A and D between groups of dogs that had been administered technical grade spinosad in gelatin capsules. In a small group of dogs in which the elimination half-life was 6.8 +/- 3 hours, 48 hours pulicidal efficacy on Day 30 was 89.1 %, compared to 48 hours efficacy of 97.8 % in dogs with slower elimination (10.6 +/- 3.7 hours) of spinosyns A and D. It was also shown that administration of oral spinosad without food markedly reduced peak plasma levels.

Orally administered spinosad can provide rapid speed of kill against existing flea infestations (Blagburn *et al*., 2010), but if efficacy is not maintained for the monthly application period, or longer if a pet owner is late in re-administering product, surviving fleas can lay eggs and perpetuate an environmental infestation (Franc & Bouhsira, 2009). In the study reported here the pioneer fipronil/(S)-methoprene topical product provided 100 % flea killing efficacy 24 hours after infestation through six weeks after treatment. Other studies have demonstrated that (S)-methoprene can provide inhibition of flea egg/larvae survival well after the adulticidal efficacy of fipronil begins to decline, providing long-lasting protection against the establishment of new environmental flea infestations (Franc *et al*., 2007; Young *et al*., 2004).

This study confirmed that providing relief to a pet and eliminating infestation of a premise by a pet requires regular dosing with formulations providing consistent and sustained activity against both adult and developmental stages of fleas.

## Footnotes

*Note:* FRONTLINE^®^ is a registered trademark of Merial. All other marks are the property of their respective owners.
